# Ultrafast green synthesis of silver nanoparticles as fluorescent nanosensors for determination of isoniazid and nitrofurantoin in biological fluids and pharmaceuticals

**DOI:** 10.1038/s41598-025-98373-6

**Published:** 2025-05-03

**Authors:** Galal Magdy, Eman Aboelkassim, Fathalla Belal

**Affiliations:** 1 Pharmaceutical Analytical Chemistry Department, Faculty of Pharmacy, Kafrelsheikh University, Kafrelsheikh, 33511 Egypt; 2 Department of Pharmaceutical Analytical Chemistry, Faculty of Pharmacy, Mansoura National University, Gamasa, 7731168 Egypt; 3 Pharmaceutical Analytical Chemistry Department, Faculty of Pharmacy, Mansoura University, Mansoura, 35516 Egypt

**Keywords:** Silver nanoparticles, Fluorescent nanosensors, Isoniazid, Nitrofurantoin, Analytical chemistry, Fluorescent probes, Sensors

## Abstract

The current study presents a green, straightforward, and ultrafast microwave-assisted method for the synthesis of Ag-NPs in only 40 s, using the *Paeonia officinalis* root extract as a reducing agent, for the first time. Characterization of the produced Ag-NPs was carried out using different spectroscopic and microscopic methods. They exhibited strong emission peak at 318 nm following excitation at 227 nm. The developed Ag-NPs were utilized as a green fluorescent nanosensor for determination of isoniazid (ISN) and nitrofurantoin sodium (NIF). The native fluorescence of Ag-NPs was observed to be quantitatively quenched by the studied drugs. Validation of the method exhibited linear relationship for ISN and NIF in the range of 20.0-100.0 µM and 10.0–60.0 µM with detection limits of 1.12 µM and 0.98 µM, respectively. Due to the high selectivity and sensitivity, the suggested method was also applied for ISN and NIF estimation in biological fluid samples and pharmaceutical formulations with acceptable % RSD and % recoveries. Assessment of the Ag-NPs’ antimicrobial activity was performed, and the synthesized Ag-NPs showed noteworthy antimicrobial activities. The evaluation of the method’s eco-friendliness and greenness was also performed using AGREE and ComplexGAPI metrics. ICHQ2 (R2) guidelines were followed to validate the developed method.

**Supplementary Information:**

The online version contains supplementary material available at 10.1038/s41598-025-98373-6.

## Introduction

*Paeonia officinalis* (common paeony) is a garden plant native to southern Europe. It is a perennial herb with red flowers and tuberous roots. Dried roots of *P. officinalis* were used medicinally in both China and India. It was used as diuretic, tonic, antispasmodic, antihypertensive, and anti-ulcer. The root was also used for treating whooping cough and epilepsy. *P. officinalis* root contains many active constituents, including paeonol, paeonin, volatile oils, tannic acid, benzoic acid, asparagin, and flavonoids^1^. All of which could reduce silver for the silver nanoparticles formation.

Silver nanoparticles (Ag-NPs) are tiny particles with nanoscale dimensions between 1 and 100 nm. Several methods could be used to synthesize Ag-NPs, including, chemical, physical, or green methods. Synthesis of Ag-NPs could be divided into two main approaches^2^. The first approach is the “top-down” approach, which depends on reducing the size of the starting materials by numerous physical methods, such as evaporation-condensation, milling, grinding, and laser ablation^3^. The second approach is the “bottom-up”, which depends on assembling smaller atoms into particles in the nano scale by chemical or green methods. In the “bottom-up” approach, reducing agents whether green or chemical are usually used to synthesize the Ag-NPs. However, the green methods, like plants^2,4^, algae^5^, fungus^6^, bacteria^7^, or irradiation-assisted methods^8^ are more favored as they are less toxic and inexpensive^9,10^. The microwave-assisted synthesis of silver nanoparticles has garnered the interest of many researchers. In contrast to traditional heating, the primary benefit of the microwave-assisted approach is its homogeneous and speedy heating process, as it does not require elevated temperatures or pressures. Consequently, employing the microwave-assisted reduction technique offers enhanced regulation over the nucleation and growth phases of nanoparticle synthesis^11,12^. The time of the procedure can be reduced. The microwave-assisted approach has superior potential owing to its expedited heating and the provision of consistent heating, resulting in evenly distributed monodispersed particles. Therefore, the microwave irradiation technique is recognized as an effective method widely employed in nanoparticle synthesis due to its more uniform process and its ability to accelerate reaction rates significantly compared to traditional heating methods^13,14^.

Silver nanoparticles are among the most prevalent metal nanoparticles utilized as advanced optical sensors for chemical, environmental, and biological investigations^2,4,8,12,15^. Compared to other metal nanoparticles, Ag-NPs may be a more advantageous choice for constructing optical sensors due to their cost-effectiveness, user-friendliness, and enhanced sensitivity. Ag-NPs have exceptional optical characteristics, including a high molar extinction coefficient, adjustable absorption wavelength, and an amplified Raman scattering effect, attributable to their unique local surface plasmon resonance (LSPR)^16,17^.

**Fig. 1:**
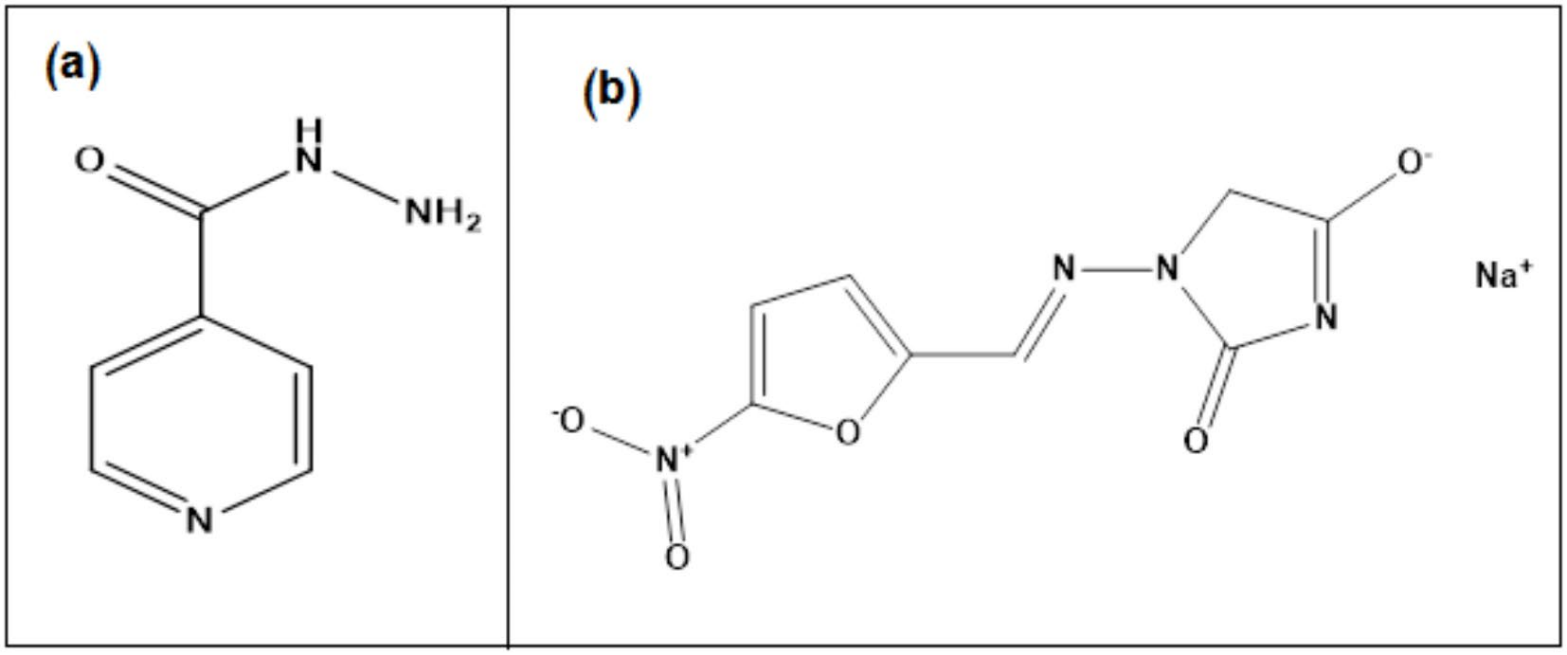
Chemical structure of ISN (**a**) and NIF (**b**).

Isoniazid (ISN) is an antibiotic drug known as isonicotinic acid hydrazide. ISN is the first line drug for preventing and treating tuberculosis^18^. It could be also used for treating latent tuberculosis, but rifampicin replaced it, since rifampicin has better completion rates and shorter treatment course^19^. ISN is a bactericidal prodrug that exerts its action by inhibiting the bacterial cell wall after activation by the catalase-peroxidase KatG^20^. The IUPAC name of ISN is pyridine-4-carbohydrazide (Fig. 1a)^21^.

Nitrofurantoin sodium (NIF) is an antibiotic for treating uncomplicated urinary tract infections (UTI). It is effective against the majority of bacteria, both gram-negative and gram-positive^22^, by preventing the growth of bacteria through converting the NIF into electrophilic intermediates that prevent the cycle of citric acid and the synthesis of protein, RNA, and DNA^23^. The use of NIF has gained interest, since it has a concurrent effect on multiple targets, making it less vulnerable to the bacterial resistance that arises as a result of the generally used antibiotics, as trimethoprim/sulfamethoxazole and quinolones^24^. The IUPAC name of NIF is sodium;3-[(E)-(5-nitrofuran-2-yl)methylideneamino]-2-oxo-4 H-imidazol-5-olate (Fig. 1b)^25^.

A variety of analytical techniques was reported for ISN determination in its pharmaceutical formulations and biological fluids, including UV spectrophotometry^26,27^, chemiluminescence^28^, spectrofluorimetry^29^, voltammetry^30^, different chromatographic techniques^31,32^, and capillary zone electrophoresis^33^. While For NIF, the reported analytical methods included spectrofluorimetry^34,35^, flow injection analysis^36^, spectrophotometry^37^, high performance liquid chromatography (HPLC)^38^, and voltammetry^39^.

The current study aims to develop an ultrafast, green, and microwave-assisted method for the Ag-NPs synthesis. The proposed method used the extract of *P. officinalis* root as a green reducing/stabilizing agent. The quenching of Ag-NPs native fluorescence by ISN and NIF was investigated and used for designing a green, quick, and straightforward method for the ISN and NIF spectrofluorimetric estimation in their biological fluids and pharmaceutical formulations. Spectrofluorimetry is acknowledged as an effective analytical technique that enhances sensitivity, selectivity, and simplicity while maintaining precision especially in comparison to other methods like as chromatography, colorimetry, and electrochemical techniques^40,41^. It is extensively accessible in many research laboratories, offering a cost-effective alternative that does not necessitate intricate apparatus or substantial quantities of dangerous chemicals, as required in methodologies such as LC-MS, GC, and HPLC^42–46^. Spectrofluorimetry, recognized for its exceptional selectivity and sensitivity, is frequently utilized to accurately quantify diverse medications in their dosage forms or biological samples^10,47–53^.

The greenness assessment of the method was studied applying the Analytical GREEnness (AGREE) and the complementary green analytical procedure index (Complex GAPI). In comparison to the reported methods for ISN and NIF estimation, although some reported methods are more sensitive than the suggested method, they have many disadvantages, like the need of highly costed devices, compelling analysis, and the time consuming procedures^29,30,38^. In contrast, the current method is green, simple, quick, and inexpensive. Therefore, it can be better suited for analysis of the studied drugs.

## Experimental

### Instruments and software

Ag-NPs synthesis was performed using domestic microwave (900-watt, Fresh Co.). The morphology of the prepared Ag-NPs was studied utilizing high resolution transmission electron microscope (HRTEM) employed at 200 kV (JEOL, Tokyo). Particle size distribution and zeta potential were detected using zeta potential and ZetaPals particle size analyzer (Brookhaven, USA). The Ag-NPs FT-IR spectrum was also recorded using Jasco 6800 Fourier transform infrared (FT-IR) spectrometer (Japan). Cary Eclipse Fluorescence Spectrophotometer from Agilent Technologies (Santa Clara, United States) was used and maintained at manual voltage 740 V. For the spectrophotometric measurements, a double beam spectrophotometer (PG Instrument, UK) was used.

### Reagents and chemicals

Isoniazid (99.98%) was generously supplied by Chemical Industries Development (CID) (Giza, Egypt) and nitrofurantoin sodium (99.95%) was supplied by Arab Company for Pharmaceutical and Medicinal Plants (MEPACO) (Cairo, Egypt). Macrofuran^®^ hard gelatin capsules (100 mg nitrofurantoin/capsule, B. No. 2220132) were purchased from local Pharmacy.

*Paeonia officinalis* root was purchased from the local market. Britton-Robinson buffer (0.02 M) covering pH ranges over 2–12 was prepared. All chemicals and reagents including boric acid, silver nitrate, methanol, glacial acetic acid, and phosphoric acid were obtained from Sigma-Aldrich (St. Louis, MO, USA).

Human urine was collected from male healthy volunteers, while human plasma samples were supplied by Kafrelsheikh University Hospital, Kafrelsheikh, Egypt and kept frozen until used at -20 ^o^C.

### General procedures

#### Preparation of P. officinalis root extract

**Fig. 2:**
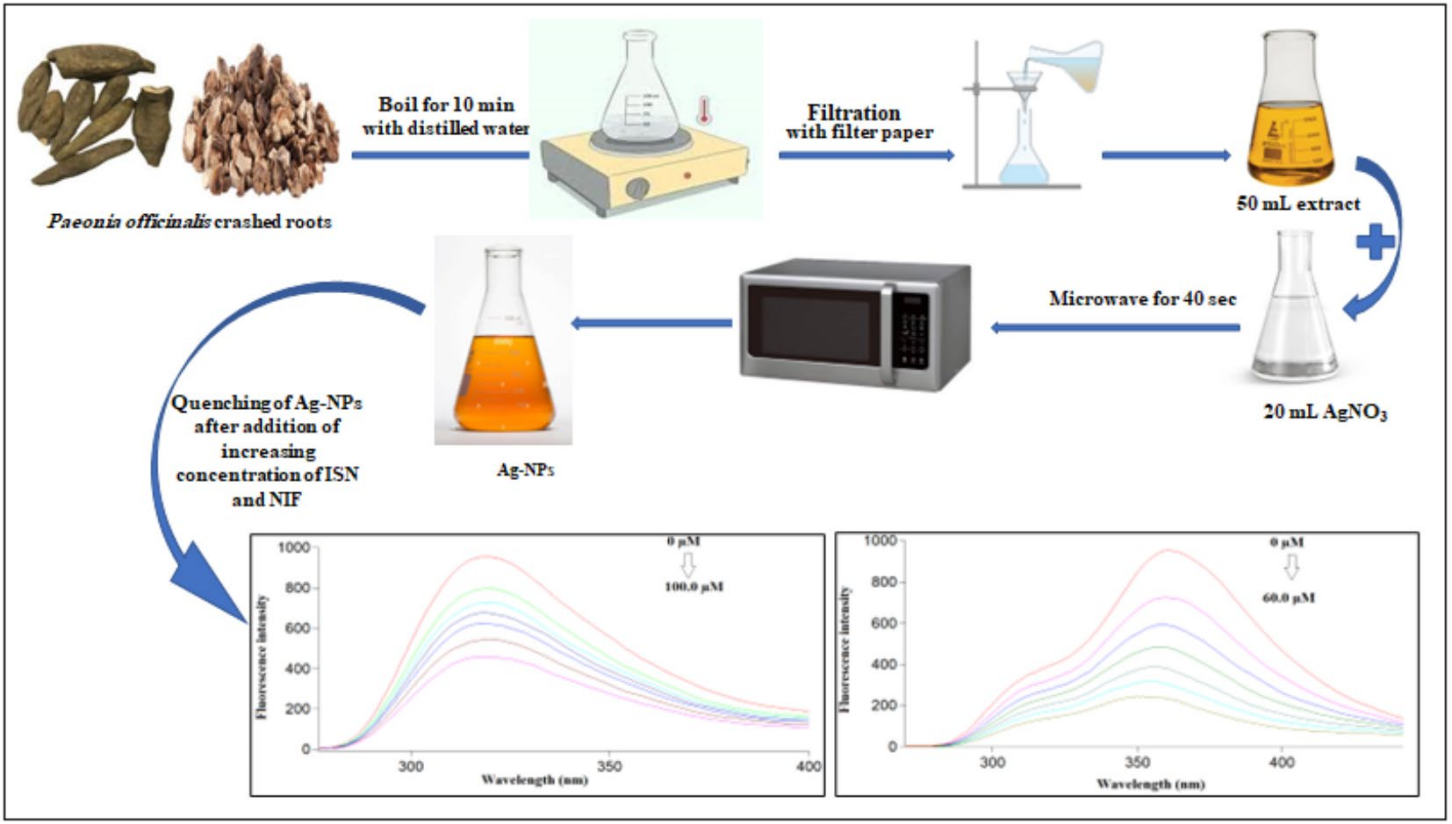
Schematic diagram for the microwave synthesis of Ag-NPs and application for the spectrofluorimetric estimation of ISN and NIF.

*P. officinalis* roots were washed to remove dust with distilled water. After drying, the roots (10 g) were crashed and boiled with distilled water (200 mL) for 10 min. Then, the aqueous extract was filtered and kept in the fridge till used (Fig. 2).

#### Microwave Preparation of Ag-NPs

Silver nanoparticles were prepared utilizing the green microwave-assisted method, by mixing 20 mL of 1.0 mM of AgNO_3_ with 50 mL of *P. officinalis* root extract before heating for 40 s in the microwave. Then, the prepared Ag-NPs solution was stored for further use in the refrigerator after cooling (Fig. 2).

#### Stock solutions of ISN and NIF

Standard solutions of ISN and NIF were separately prepared after dissolving 0.0069 g and 0.013 g, respectively, in 50 mL of methanol to give a stock solution of 1.0 mM concentration. Using distilled water, subsequent dilution of the standard solutions was carried out for preparing working solutions covering the concentration range of 20.0-100.0 µM and 10.0–60.0 µM for ISN and NIF, respectively. ISN and NIF standard solutions remained stable for minimum a month upon keeping in the fridge.

#### Calibration graphs

After optimizing the experimental parameters, in a set of 10 mL volumetric flasks, varying aliquots of ISN, in the range of 20.0–100.0 µM, were added to 100.0 µL of Ag-NPs and the flasks were completed to the mark with distilled water. For NIF, 1.0 mL of Britton-Robinson buffer of pH 10 was added to 100.0 µL of Ag-NPs followed by aliquot volumes of NIF in the range of 10.0–60.0 µM, and the flasks were completed with distilled water to 10.0 mL.

All measurements were done at ambient temperature and fluorescence intensities were measured at 318 nm after excitation at 227 nm. For regression analysis, calibration curves were constructed through plotting the fluorescence quenching by ISN and NIF vs. their concentrations in µM.

#### Analysis of commercial dosage forms

A number of laboratory-prepared tablets of ISN (Isocid^®^) were prepared by mixing with magnesium stearate (10 mg), maize starch (15 mg), lactose (15 mg), and talc (20 mg) for each tablet, while maintaining the pharmaceutical concentration of the drug. An amount of powder corresponding to 6.86 mg was weighed and mixed with 20 mL of methanol. Then, the mixture was sonicated for 10 min, filtered into a 50-mL volumetric flask, and completed with methanol to the mark giving a final concentration of 1.0 mM. For NIF, ten capsules of Macrofuran^®^ (100 mg nitrofurantoin / capsule) were individually emptied, thoroughly mixed, and weighed. An amount of powder corresponding to 12 mg of NIF was weighed before mixing with 20 mL of methanol. Then, the mixture was sonicated for 10 min, filtered into a 50-mL volumetric flask, and completed to the mark with methanol to give a final concentration of 1.0 mM.

In 10-mL volumetric flasks, subsequent dilution was made with distilled water to get the working solutions. Finally, the procedure previously mentioned in Section “**2.3.4.**” was employed, and to measure the nominal content, the corresponding regression equations were used.

#### Analysis of ISN in plasma samples

In a set of 15 mL centrifugal tubes, aliquots of ISN stock solutions were added to 1 mL of drug-free plasma, followed by plasma deproteination by completing to 5 mL with methanol. The mixtures were vortex mixed for 2 min before centrifugation at 9000 rpm for 10 min. Into a set of 10 mL volumetric flasks, 1 mL of filtered supernatant was added to 100.0 µL of Ag-NPs and completed by distilled water to the mark. Final concentrations werre adjusted over the range of 25.0–50.0 µM ISN, and fluorescence measurements were made in accordance with section “**2.3.4.**” with a blank experiment conducted in parallel, then the corresponding regression equation was derived. All methods were performed in accordance with relevant guidelines and regulations, and all experimental protocols were approved by the Committee of Research Ethics in the Faculty of Pharmacy, Kafrelsheikh University, Kafrelsheikh, Egypt.

#### Analysis of NIF in urine samples

Into a set of 15 mL centrifugal tubes, aliquots of NIF stock solutions were add to 1 mL aliquots of drug-free urine, followed by completing to 5 mL with methanol. The mixtures were vortex mixed for 2 min before centrifugation at 9000 rpm for 10 min. Into a set of 10 mL volumetric flasks, 1 mL of filtered supernatant was added to 1 mL of Britton-Robinson buffer (pH 10) and 100.0 µL of Ag-NPs. The solution was completed by distilled water to the mark, and final concentration was adjusted over the range of 10.0–60.0 µM. Fluorescence measurements were made in accordance with section “**2.3.4.**” with a blank experiment conducted in parallel, then the corresponding regression equation was derived. All methods were performed in accordance with relevant guidelines and regulations, and all experimental protocols were approved by the Committee of Research Ethics in the Faculty of Pharmacy, Kafrelsheikh University, Kafrelsheikh, Egypt.

#### Antimicrobial study

For studying the antimicrobial activity, AgNO_3_, *P. officinalis* root extract, and Ag-NPs were separately verified against a panel of Gram positive and negative bacteria, involving *S. aureus*, *B. subtilis P. aeruginosa*, and *E. coli*. Their anti-fungal activities were also verified against *Candida albicans*. Utilizing the agar well diffusion method, 100.0 µl of sample solution were added to a well containing nutrient agar media of peptone (5 g), beef extract (3 g), and agar (20 g) after seeding with the tested bacteria and fungi. Finally, recording of the inhibition zones was performed after petri dishes incubation for 24 h at 36 ^o^C.

#### Measurement of fluorescence quantum yield

The quantum yield of Ag-NPs was computed utilizing the subsequent Equation ^54^:

$$\:\varvec{\varPhi\:}\text{Ag-NPs}\:\:=\:\:\varvec{\varPhi\:}\text{St}\:\times\:\left.\left(\frac{\varvec{F}\text{Ag-NPs}\:}{\varvec{F}\text{St}}\right.\right)\times\:\left.\left(\frac{\varvec{A}\text{St}\:}{\varvec{A}\text{Ag-NPs}}\right.\right){\times\:\left.\left(\frac{\varvec{\eta\:}\text{Ag-NPs}}{\varvec{\eta\:}\text{St}}\right.\right)}^{2}$$

In this context, Φ denotes the quantum yield, F signifies the integrated fluorescence intensity of Ag-NPs and the standard (St) following excitation at 227 nm, A represents the absorbance at 227 nm, and η indicates the refractive index of the solvent (η = 1.33 for both solvents). A 0.1 M H_2_SO_4_ solution of 2-amino pyridine served as the standard, with Φ_St_ equal to 0.6. The absorbance values (A_St_ and A_Ag−NPs_) were maintained below 0.1 to mitigate the absorption effect.

## Results and discussion

### Synthesis and characterization of Ag-NPs

**Fig. 3:**
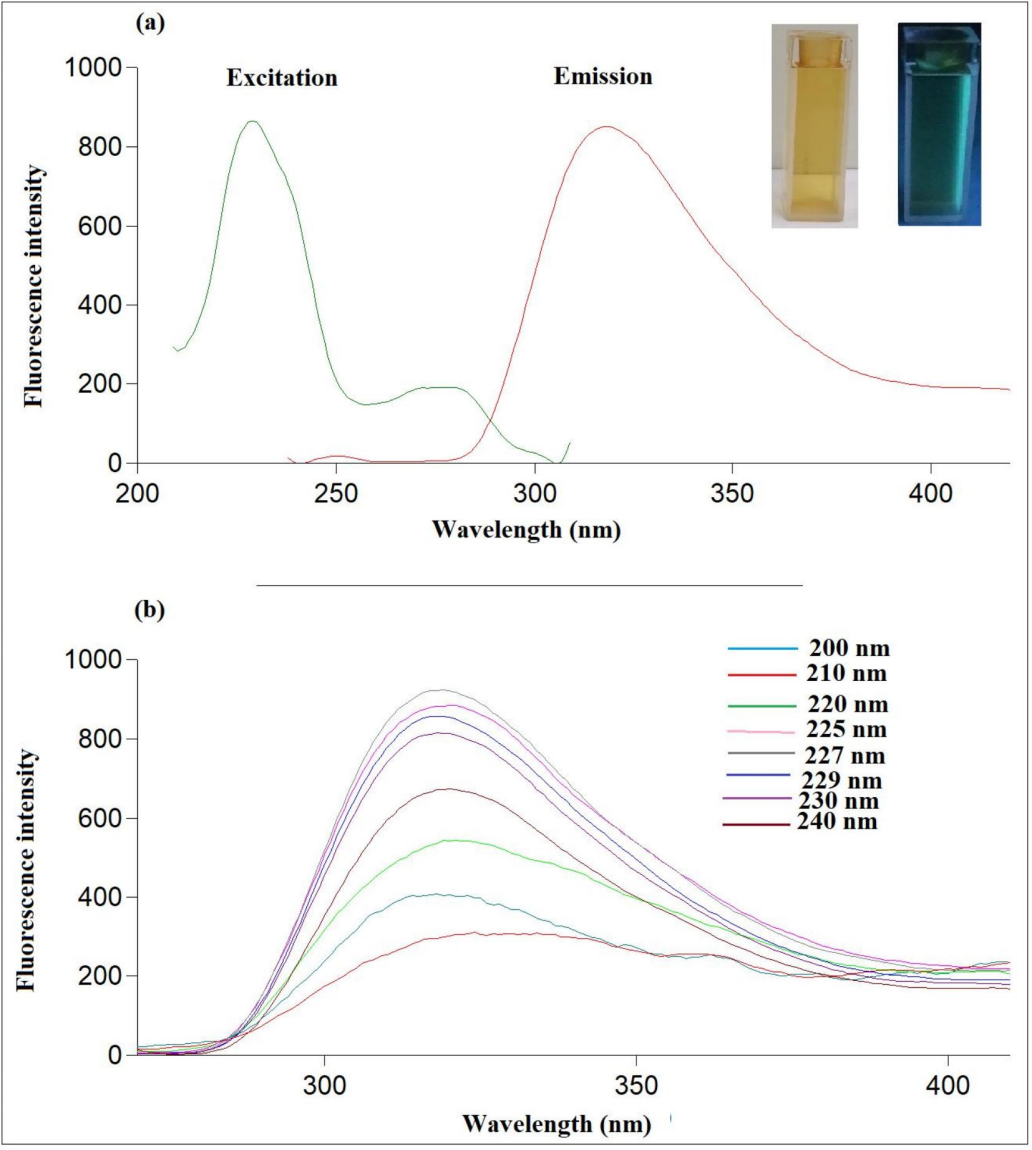
(**a**) Fluorescence emission spectra of Ag-NPs at 318 nm after excitation at 227 nm (inset: photograph of Ag-NPs under visible light and UV light), (**b**) Fluorescence emission spectra of the Ag-NPs at different excitation wavelengths (200–240 nm).

Here in, a green, simple, and quick method was used for Ag-NPs synthesis by the microwave in only 40 s using *P. officinalis* root extract for the first time (Fig. 2). The produced Ag-NPs revealed blue fluorescence after exposing to UV light and dark brown color under daylight (Fig. 3).

Different techniques, including TEM, FT-IR, spectrofluorimetry, and UV-vis spectroscopy were applied to characterize the synthesized Ag-NPs. Figure S1 shows the AgNO_3_ and Ag-NPs absorption spectra. The synthesized nanoparticles and AgNO_3_ were observed to have a distinct UV absorption peak at 237 nm and 219 nm, respectively. Fluorescence properties of Ag-NPs were also investigated. Figure 3a revealed the fluorescence spectrum of the prepared nanoparticles, founding that they have high fluorescence intensity at 318 nm following excitation at 227 nm. The emission of the developed Ag-NPs showed excitation dependency across the range of 200–240 nm, and the highest emission was obtained at 227 nm (Fig. 3b). The quantum yield of Ag-NPs was also assessed using 2-amino pyridine as a reference, yielding an ideal quantum yield of around 43%.

Particle size distribution and zeta potential were also measured for the prepared Ag-NPs, revealing that their average diameter and polydispersity was 47.4 nm and 0.247, respectively, while Zeta potential was − 14.85 mV (Fig. S2).

**Fig. 4:**
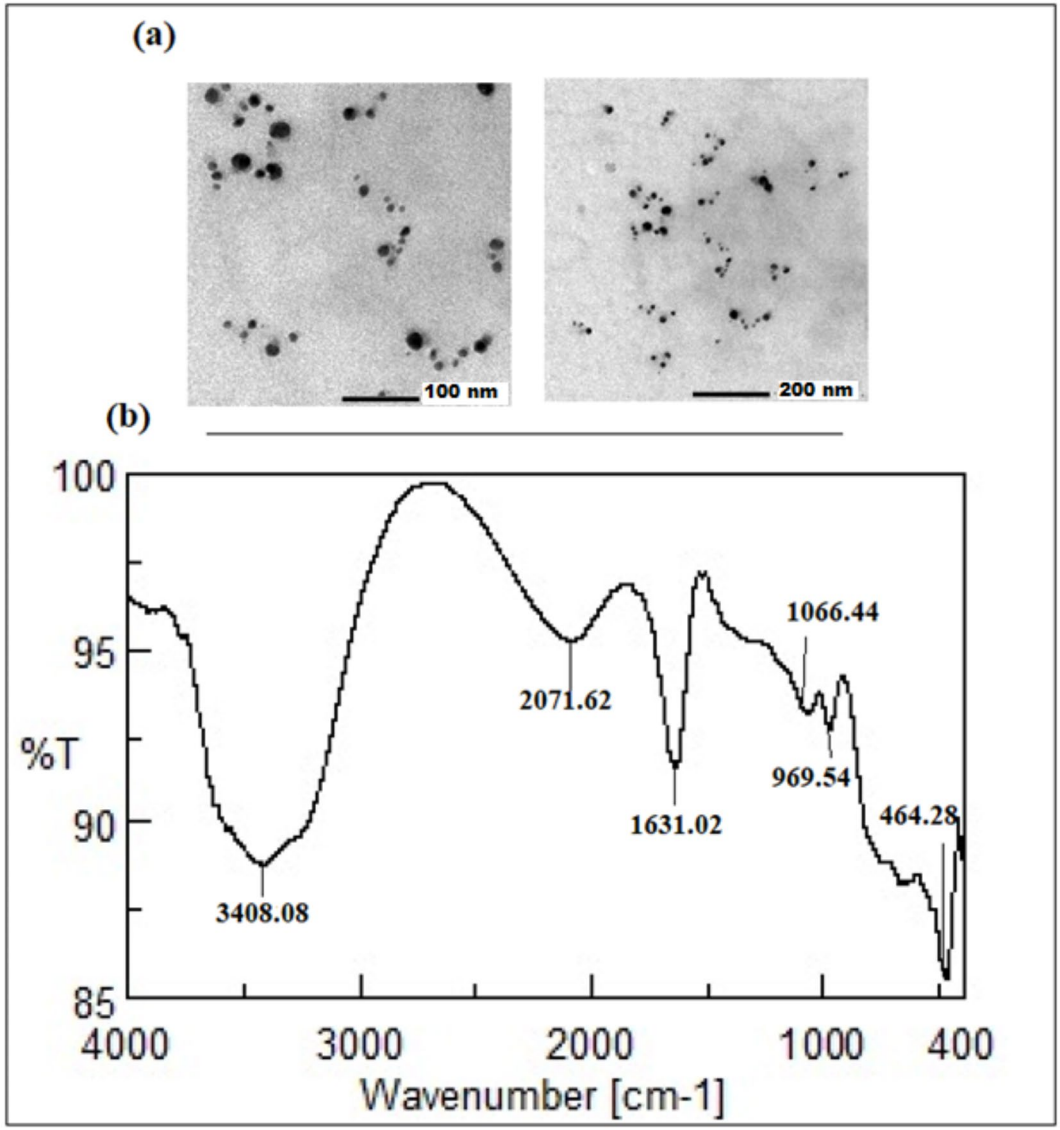
(**a**) the typical TEM images of Ag-NPs, (**b**) FT-IR spectra of synthesized Ag-NPs.

Moreover, HRTEM was used to display the surface morphological features of the prepared nanoparticle, and they were found to be spherical, well separated, and homogenous (Fig. 4a). Finally, FT-IR spectrometer was utilized to determine the developed nanoparticles surface functional groups, demonstrating stretching peaks of N-H at 3408 cm^− 1^, C-N at 2071 cm^− 1^, C = C at 1631 cm^− 1^, C-O at 1066 cm^− 1^, C-H at 969 cm^− 1^, and C-I at 464 cm^− 1^ (Fig. 4b)^55^.

### Investigation of the quenching mechanism

**Fig. 5:**
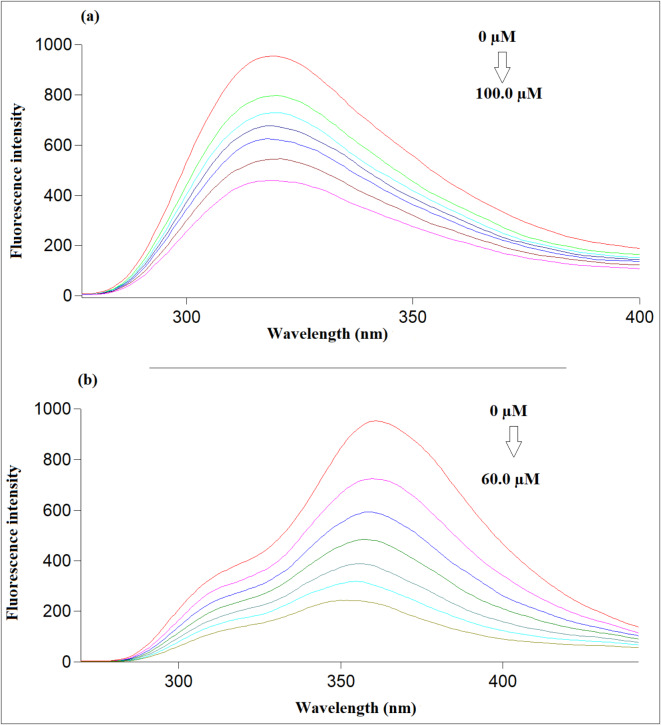
Fluorescence emission spectra of Ag-NPs upon addition of different concentrations of (**a**) ISN (from top to bottom: 0, 20.0, 40.0, 50.0, 60.0, 80.0, 100.0 µM), (**b**) NIF (from top to bottom: 0, 10.0, 20.0, 30, 40.0, 50.0, 60.0 µM).

In the current study, Ag-NPs were synthesized and used for the spectrofluorimetric determination of ISN and NIF. The Ag-NPs fluorescence intensity was decreased upon adding increasing concentration of each ISN and NIF, as shown in Fig. 5.

**Fig. 6:**
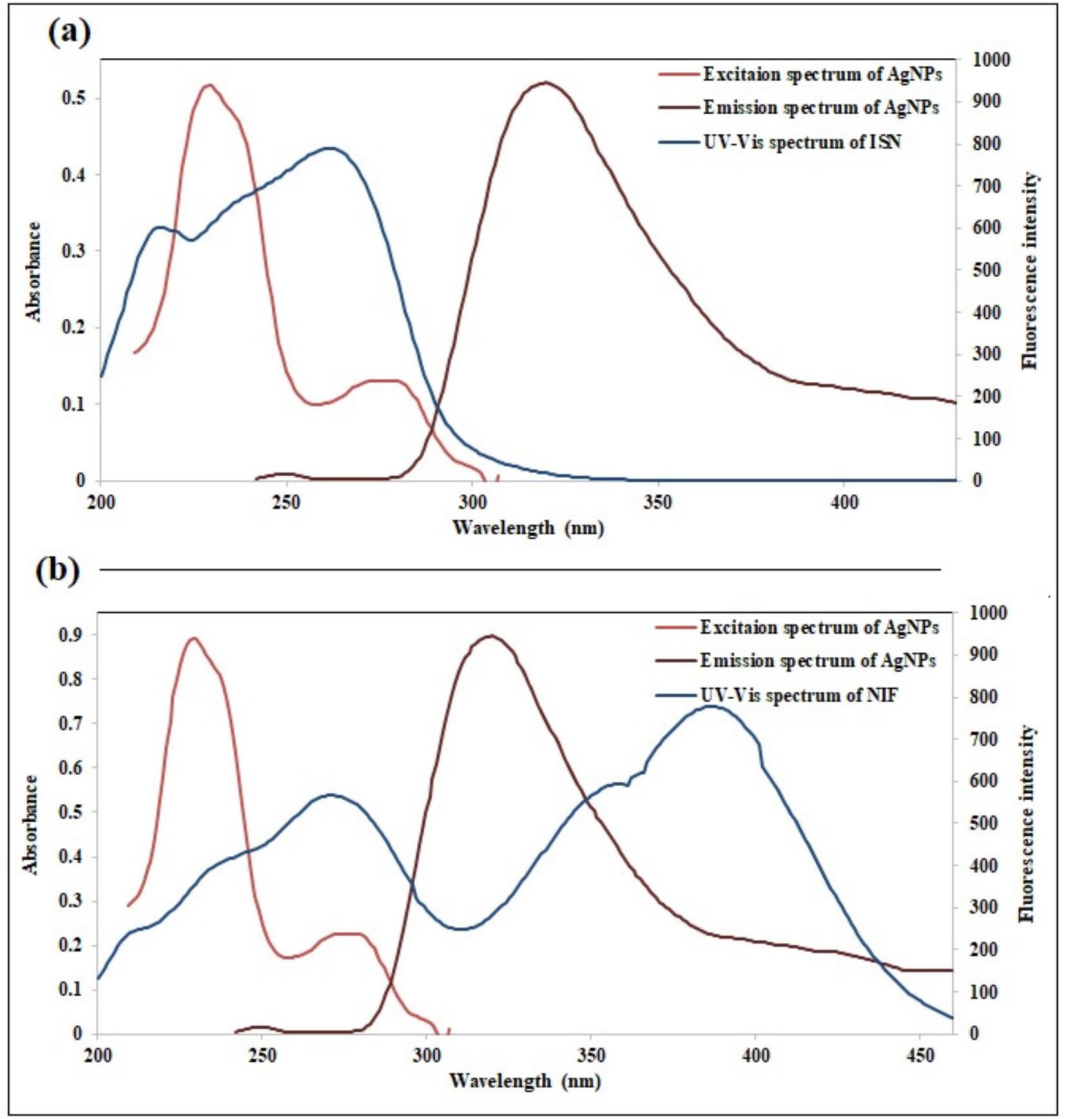
A co-plot showing the overlapping between the fluorescence excitation and emission spectra of Ag-NPs and the UV-Vis absorption spectrum of ISN (**a**) and NIF (**b**).

Generally, the term “quenching” refers to any decrease in the fluorescent probe’s intensity. Quenching mechanisms of nanoparticles mainly include dynamic and static quenching, along with inner filter effect (IFE)^56–61^. IFE occurs once the quencher (ISN or NIF) has absorption at the excitation or emission spectra of the nanoparticles^42,62,63^. As demonstrated in Fig. 6, the ISN and NIF absorption spectra were greatly overlapped with the excitation spectrum of Ag-NPs. Also, there is an overlap between NIF absorption spectrum and the emission spectrum of Ag-NPs, consequently IFE could potentially take place.

Further study on the quenching mechanism was carried out by correcting the intensity of Ag-NPs fluorescence for any potential IFE following adding increasing concentrations of the aforementioned drugs (ISN or NIF), using the following Eq. (1):

1 $$\:{\varvec{F}}_{\varvec{c}\varvec{o}\varvec{r}\varvec{r}}=\:\:\:{\varvec{F}}_{\varvec{o}\varvec{b}\varvec{s}}\times\:{10}^{\left(\frac{{A}_{ex}+{A}_{em}}{2}\right)}$$

Where: **F**_**obs**_ and **F**_**corr**_ are the observed and corrected fluorescence, **A**_**em**_ is the ISN or NIF absorbance at the emission wavelength and **A**_**ex**_ is their absorbance at the excitation wavelength.

In addition, the suppressed efficiency (**%E**) of the corrected and observed fluorescence was defined in accordance to Eq. (2):

2 $$\:\varvec{\%}\varvec{E}=[1-\left(\frac{\varvec{F}}{{\varvec{F}}_{0}}\right)]\times\:100$$

Where: **F**_**0**_ refers to the fluorescence intensity in the absence of the quencher. **F** is the **F**_**obs**_ or **F**_**corr.**_

**Fig. 7:**
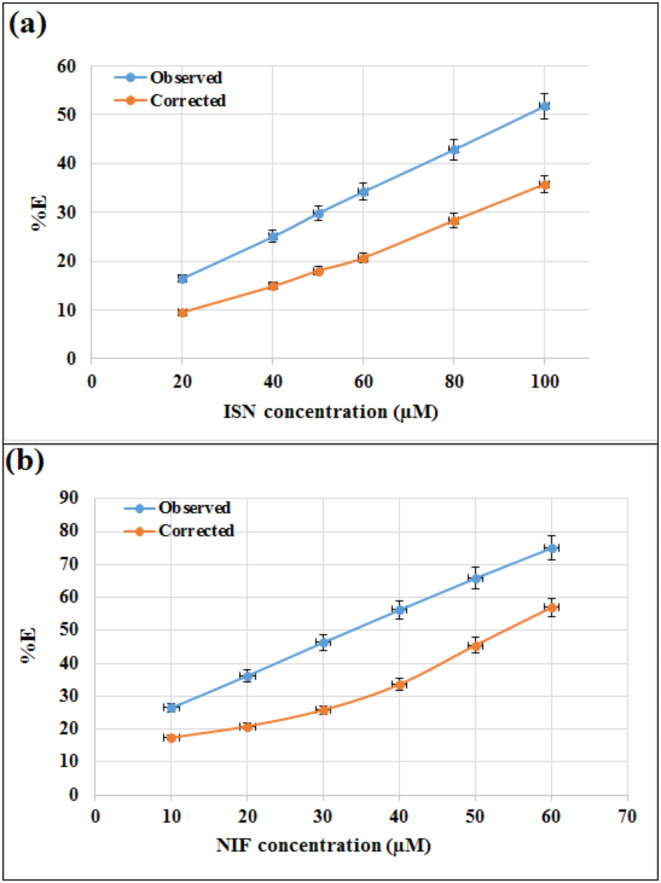
%E of observed and corrected fluorescence of Ag-NPs after the addition of various concentrations of (a) ISN (20.0, 40.0, 50.0, 60.0, 80.0, 100.0 µM), (b) NIF (10.0, 20.0, 30, 40.0, 50.0, 60.0 µM).

The plot of **%E** for ISN and NIF concentrations (µM) vs. Ag-NPs observed and corrected fluorescence intensities revealed that, IFE have a prominent role in the quenching of Ag-NPs by ISN and NIF (Fig. 7), which may be primary and secondary IFE for ISN and NIF, respectively.

For dynamic and static quenching investigation, Stern-Volmer Eq. (3) was employed as following^10,64^:

3 $$\:\frac{{\varvec{F}}_{0}}{\varvec{F}}=1+{\varvec{K}}_{\varvec{s}\varvec{v}}\left[\varvec{Q}\right]$$

Where: [Q] represents the molar concentration of ISN or NIF, K_SV_ is the constant of Stern-Volmer, and (F_0_) and (F) are the Ag-NPs fluorescence intensities in the cited drugs absence and presence^65^.

**Fig. 8:**
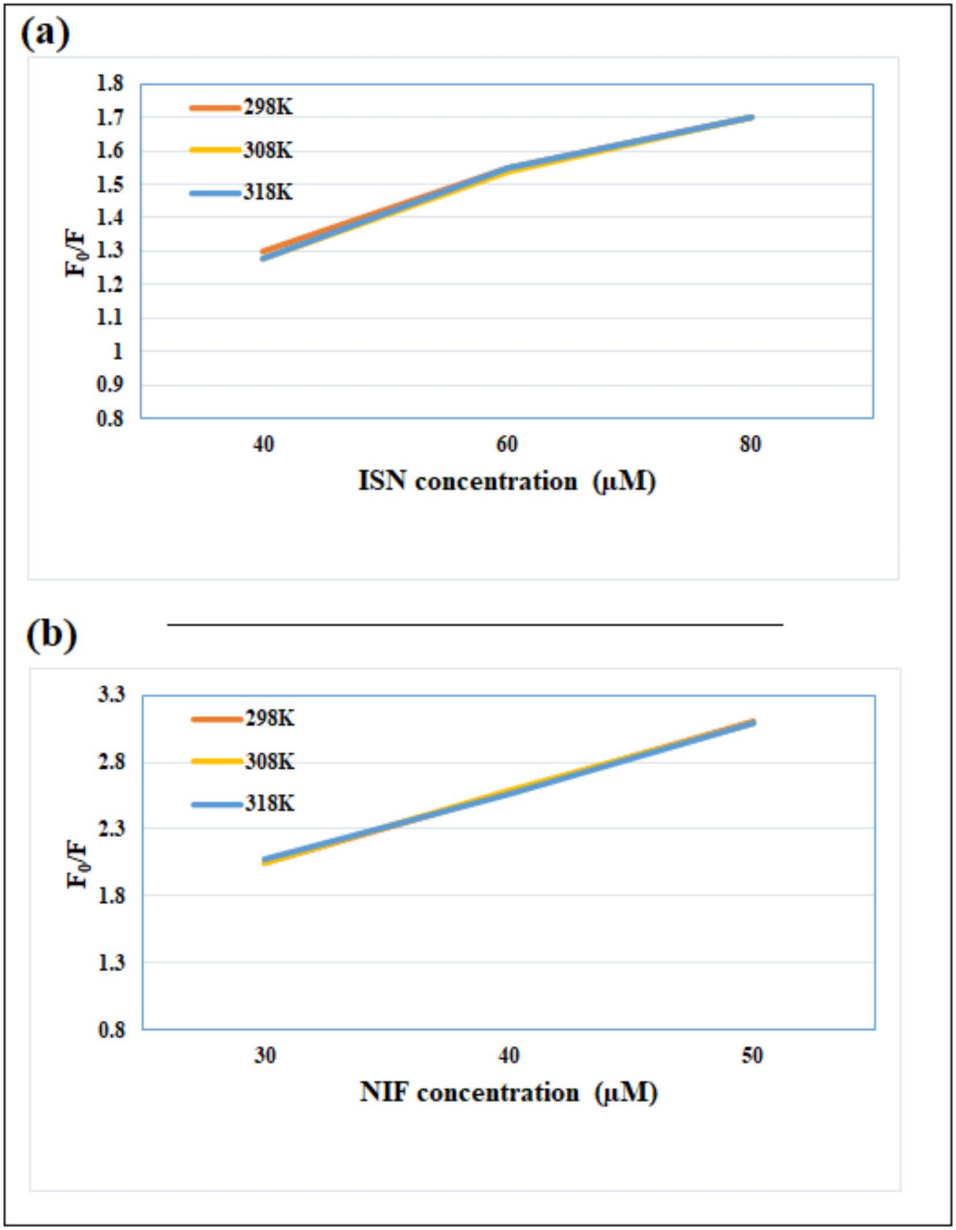
Stern-Volmer plots for the quenching of Ag-NPs fluorescence at three different temperature settings (298, 308, and 318 K) by different concentrations of (a) ISN (40.0, 60.0, 80.0 µM), (b) NIF (30.0, 40.0, 50.0 µM).

For investigating the mechanisms of quenching complementary with IFE, the Stern-Volmer plot between the F_0_/F and the quencher concentration at 298 K, 308 K, and 318 K was performed, and the K_SV_ constant were measured (Fig. 8)^66–68^. For ISN, the K_SV_ values were found to be 8.93 × 10^3^, 8.91 × 10^3^, and 8.96 × 10^3^, while for NIF, they were 41.26 × 10^3^, 41.14 × 10^3^, and 41.04 × 10^3^ at 298, 308, 318 K, respectively. As illustrated the K_SV_ almost was not affected by the raising of temperatures, therefore dynamic and static quenching mechanisms were excluded.

### Optimization of experimental parameters

#### pH effect

**Fig. 9:**
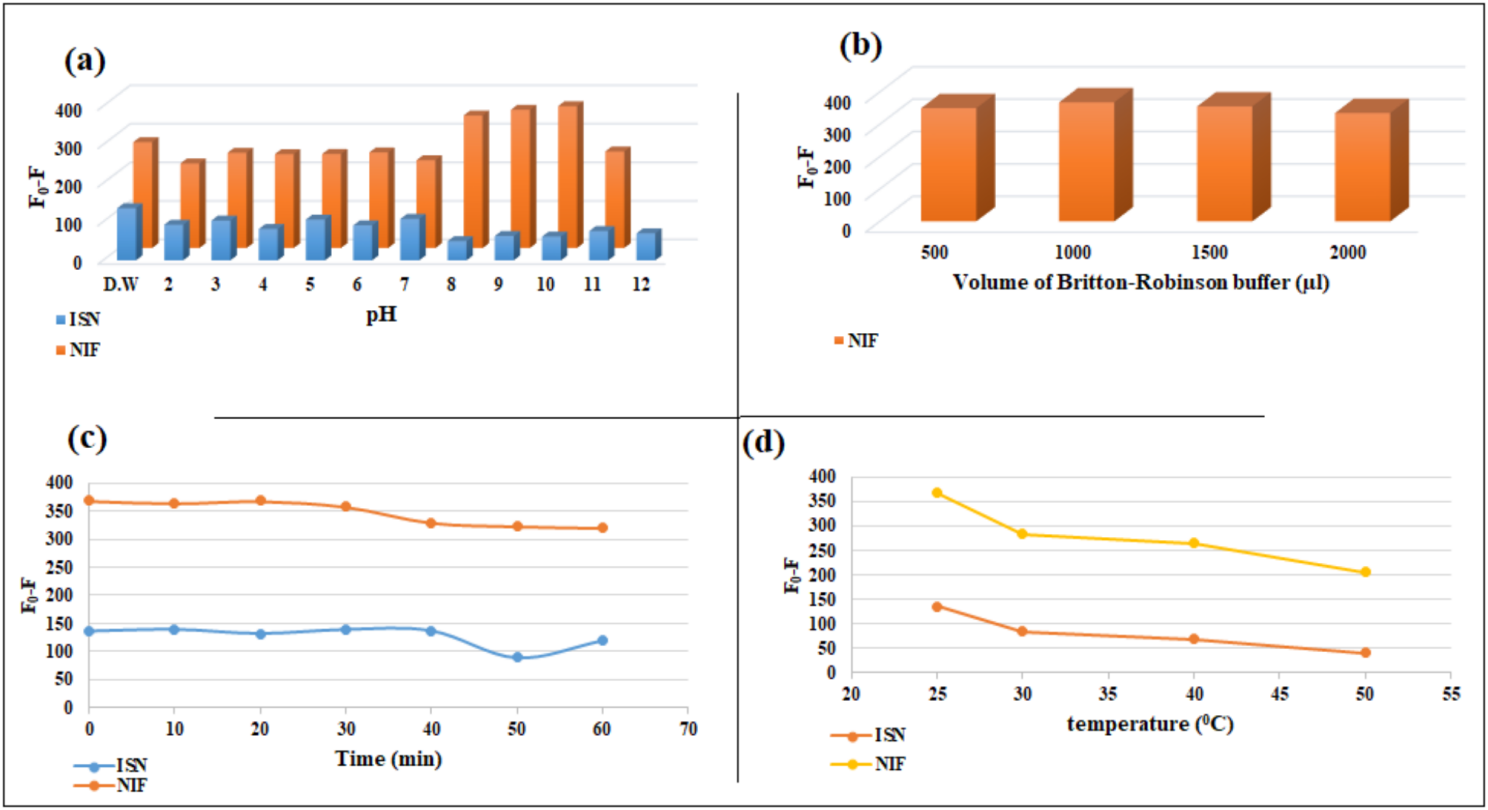
Effect of pH (**a**), volume of Britton-Robinson buffer (**b**), incubation time (**c**), and temperature (**d**) on the relative quenched fluorescence intensity of Ag-NPs by 20.0 µM of each of ISN and NIF.

Britton-Robinson buffer with pH range from 2 to 12 was used to study the pH effect on Ag-NPs fluorescence quenching by either ISN or NIF. For ISN, there was insignificant difference in the quenching of Ag-NPs over the pH range, while for NIF, it was found that the ideal one is pH 10 (Fig. 9a).

#### Buffer volume effect

Effect of buffer volume for NIF was investigated over the volume range of 0.5–2.0 mL, revealing that, 1.0 mL of buffer resulted in the highest quenching of Ag-NPs fluorescence (Fig. 9b).

#### Time effect

Time effect was investigated at different intervals between 1 and 60 min, founding that Ag-NPs quenching by ISN and NIF was instantaneous and stable for at least 1 h (Fig. 9c).

#### Temperature effect

Effect of temperature was also investigated for both ISN and NIF from 25 °C to 50 °C. It was found that Ag-NPs quenching by ISN and NIF greatly diminished by raising the temperature (Fig. 9d). Consequently, the spectrofluorimetric measurements were made at room temperature.

### Method validation

Method validation was performed in accordance with ICHQ2 (R2) guidelines^69^.

#### Linearity and range

Under optimized conditions, the quenching of Ag-NPs fluorescence by ISN and NIF was recorded, and the calibration curve was plotted using the final ISN or NIF concentration (µM) vs. the fluorescence quenching (F_0_-F). The ISN and NIF linear range was 20.0-100.0 µM and 10.0–60.0 µM, respectively. Linear regression analysis could be represented by the given equations:

4 $$\left( {{{\bf{F}}_{\bf{0}}} - {\bf{F}}} \right){\rm{ }} = {\rm{ }}{\bf{4}}.{\bf{218}}{\rm{ }}{\bf{C}}{\rm{ }} + {\rm{ }}{\bf{72}}.{\bf{33}}\;\;\;\;\;\;\;\;\;\;\;\;\;\;\;\;\;\;\left( {{\bf{r}}{\rm{ }} = {\rm{ }}{\bf{0}}.{\bf{9999}}} \right){\rm{ }}{\bf{for}}{\rm{ }}{\bf{\,ISN}}$$

5 $$\left( {{{\bf{F}}_{\bf{0}}} - {\bf{F}}} \right){\rm{ }} = {\rm{ }}{\bf{9}}.{\bf{464}}{\rm{ }}{\bf{C}}{\rm{ }} + {\rm{ }}{\bf{164}}.{\bf{31}}\;\;\;\;\;\;\;\;\;\;\;\;\;\;\;\;\left( {{\bf{r}}{\rm{ }} = {\rm{ }}{\bf{0}}.{\bf{9999}}} \right){\rm{ }}{\bf{for\,}}{\rm{ }}{\bf{NIF}}$$

Where: (F_0_) and (F) represent the fluorescence intensities of Ag-NPs with or without the studied drugs, C is the concentration (µM) of the cited drugs, and r refers to the correlation coefficient. The analytical method performance data for ISN and NIF estimation was demonstrated in Table 1.

**Table 1:** Analytical performance data of the proposed method.

Parameters	ISN	NIF
Linear concentration range (µM)	20.0-100.0	10.0–60
LOD (µM)	1.12	0.98
LOQ (µM)	3.40	2.97
Slope	4.218	9.464
Intercept	72.33	164.31
Correlation coefficient (r)	0.9999	0.9999
S.D. of the residuals, S_y/x_	1.43	3.02
S.D. of the intercept, S_a_	1.43	2.81
S.D. of the slope, S_b_	0.02	0.07
Percentage relative standard deviation (% RSD)	0.70	1.02
Percentage relative error (% Error)	0.28	0.42

#### Detection (DL) and quantitation limits (QL)

DL and QL can be determined according to the following Eq. ^70^:

6 $$\:\varvec{L}\varvec{O}\varvec{D}=3.3\frac{{\varvec{S}}_{\varvec{a}}}{\varvec{b}}$$

7 $$\:\varvec{L}\varvec{O}\varvec{Q}=10\frac{{\varvec{S}}_{\varvec{a}}}{\varvec{b}}$$

Where, S_a_ is the intercept’s standard deviation and b is the calibration curve’s slope. The acquired values of DL and QL proved that the current method is sensitive enough for estimating ISN and NIF in biological fluids (Table 1).

#### Accuracy and precision

Method accuracy was studied by comparing the current method data with the obtained data by the comparison techniques^27,34^. Table 2 demonstrated that there was negligible variation between the calculated and tabulated values of t-test and the variance ratio F-test, demonstrating the high accuracy and precision of the suggested method. Moreover, the method precision was studied and both ISN and NIF showed small %RSD values (≥ 0.74%), as shown in Table S1.

**Table 2:** Application of the proposed method for the determination of the studied drugs in Raw materials.

Parameter	ISN			NIF		
	Conc. taken (µM)	Conc. found (µM)	% recovery^a^	Conc. taken (µM)	Conc. found (µM)	% recovery^a^
	20.0	20.10	100.52	10.0	9.82	98.25
	40.0	39.50	98.75	20.0	19.84	99.21
	50.0	50.26	100.51	30.0	30.23	100.78
	60.0	60.32	100.53	40.0	40.31	100.77
	80.0	79.81	99.77	50.0	50.20	100.39
	100.0	100.00	100.01	60.0	59.60	99.33
**Mean**			100.02			99.79
**± SD**			0.70			1.02
**% RSD**			0.70			1.02
**% Error**			0.28			0.42
	**Comparison method** ^27^			**Comparison method** ^34^		
**Mean ± SD**	99.32 ± 1.29			99.71 ± 1.72		
**N** ^**c**^	3			3		
**t-value**	0.41 (2.78)^b^			0.24 (2.78)^b^		
**F-value**	2.07 (19.00)^b^			1.81 (19.00)^b^		

^a^ Mean of three determinations.

^b^ Values in parenthesis are the tabulated t- and F- values at *p* = 0.05 ^70^.

^c^ Number of samples.

#### Method selectivity

**Fig. 10:**
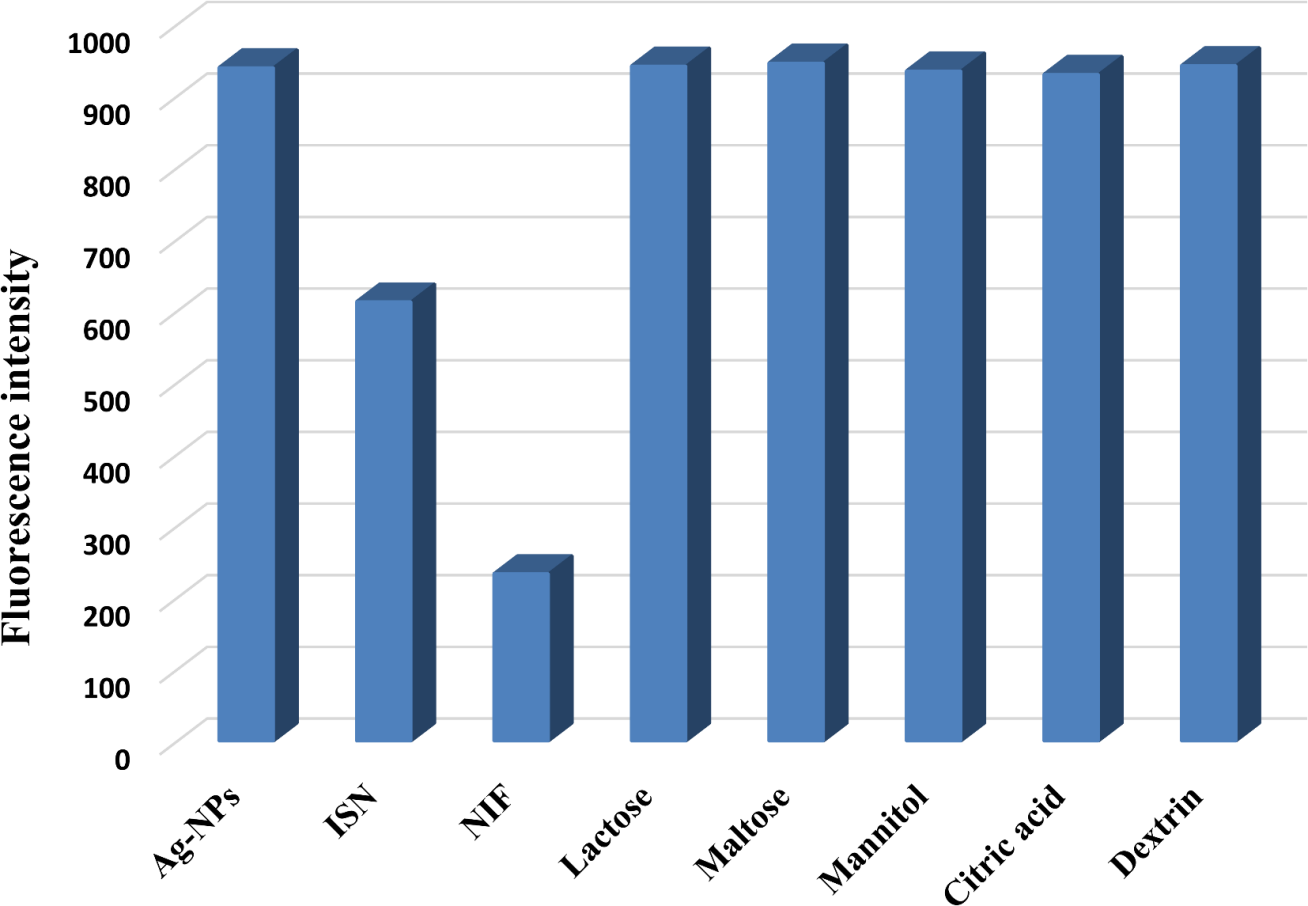
The selectivity of Ag-NPs towards ISN and NIF in presence of 1000.0 µM of common excipients.

Method selectivity for the determination of ISN and NIF was studied, and the developed method could determine the aforementioned drugs in their commercial dosage forms with high % recoveries (98.55-100.63%) and %RSD values ≥ 1.02% **(**Table 3**)**. In order to confirm the method selectivity, common excipients found in tablets were thoroughly examined in order to rule out any potential interference. None of the investigated excipients significantly quenched the fluorescence of Ag-NPs, even at an excess concentration of 1000.0 µM, which is more than 15-folds of the used ISN and NIF concentration (60.0 µM) (Fig. 10). In addition, different antimicrobial drugs were tested, and it was found that the method could determine ISN and NIF without interference from clindamycin and clarithromycin, as these drugs did not quench the Ag-NPs fluorescence. Moreover, the co-administrated drugs, rifampicin and cephalexin were tested, and the tolerance limits were measured. The tolerance limits of rifampicin and cephalexin were 0.1 and 3.0 µM, respectively. The developed method could also be applied for the NIF and ISN determination in biological fluids like spiked human urine and plasma, respectively. Application on biological fluids demonstrated high % recoveries (98.40-101.66%) and low %RSD values (1.41%) **(**Tables 4 and 5**)**, establishing the excellent selectivity of the suggested method.

**Table 3:** Application of the proposed method for the determination of the studied drugs in commercial dosage forms.

Parameter	Prepared ISN tablets		
	Conc. Taken (μM)	Conc. Found (μM)	% recovery^a^
	40.0	39.91	99.78
	60.0	60.38	100.63
	80.0	78.88	98.61
Mean			99.67
± SD			1.02
% RSD			1.02
	**Comparison method ** ^27^		
Mean ± SD	99.31 ± 1.81		
N^c^	3		
t-value	0.30 (2.78)^b^		
F-value	3.15 (19.00)^b^		
Parameter	**Macrofuran** ^**®**^ ** hard gelatin capsules (nitrofurantoin 100 mg / tablet)**		
	**Conc. Taken (μM)**	**Conc. Found (μM)**	**% recovery** ^a^
	10.0	9.89	98.92
	20.0	19.71	98.55
	30.0	30.13	100.43
Mean			99.3
± SD			1.00
% RSD			1.00
	**Comparison method ** ^34^		
Mean ± SD	100.31 ± 1.91		
N^c^	3		
t-value	0.82 (2.78)^b^		
F-value	3.67 (19.00)^b^		

^a^ Mean of three determinations.

^b^ Values in parenthesis are the tabulated t- and F- values at *p* = 0.05 ^70^.

^c^ Number of samples.

**Table 4:** Application of the proposed method for the determination of ISN in spiked human plasma.

Parameter	Conc. Taken (µM)	Conc. Found (µM)	% recovery^a^
	25.0	25.41	101.66
	35.0	34.44	98.40
	40.0	39.81	99.52
	50.0	50.34	100.68
**Mean**			100.07
**± SD**			1.41
**% RSD**			1.41
**% Error**			0.71

^a^ Mean of three determinations.

**Table 5:** Application of the proposed method for the determination of NIF in spiked human urine.

Parameter	Conc. Taken (µM)	Conc. Found (µM)	% recovery^a^
	30.0	29.61	98.69
	40.0	40.43	101.06
	50.0	50.32	100.65
	60.0	59.64	99.40
**Mean**			99.95
**± SD**			1.10
**% RSD**			1.10
**% Error**			0.55

^a^ Mean of three determinations.

#### Robustness

Robustness was evaluated by making minor differences to find out if these changes significantly affected the quenching of nanoparticles fluorescence by the aforementioned drugs. For NIF, changes were the volume of Ag-NPs (100.0 µL ± 5), volume of Britton-Robinson buffer (1 mL ± 0.1), and its pH (10 ± 0.1). While for ISN, they were only the volume of Ag-NPs (100.0 µL ± 5). It was verified that changes made to the experimental conditions had a little impact on the ISN and NIF quenching of the prepared Ag-NPs, which indicated the method’s robustness (**Table S2)**.

### Method applications

#### ISN and NIF analysis in pharmaceutical dosage forms

ISN and NIF were efficiently assayed in their pharmaceutical preparations including, prepared Isocid^®^ tablets and Macrofuran^®^ hard gelatin capsules for ISN and NIF, respectively, without any interference from excipients. Percent recovery was found to be 98.55-100.63% and the %RSD values were less than 1.02%. Data obtained from the suggested method were compared to the data of the comparison techniques^27,34^. As shown in Table 3, there was an insignificant difference between the calculated and tabulated values of variance ratio F-test and t-test, demonstrating the high precision and accuracy of the developed method.

#### Assessment of ISN in human plasma

The developed method was successfully applied for the determination of ISN in spiked human plasma samples as the method’s sensitivity could reach its peak plasma concentration (C_max_), where its linear concentration range was 20.0-100.0 µM, and its peak plasma concentration was reported to be in the range of 3.0–7.0 µg/mL (22.0–51.0 µM) after oral dose of 300 mg^70^. Upon plotting the concentrations (µM) of ISN vs. the fluorescence quenching (F_0_-F), linear relationship was achieved in the ISN spiked plasma samples. After data statistical analysis, the mean % recoveries ± SD of ISN in the samples of plasma were 100.07% ± 1.41 (Table 4, Fig. S3). Linear regression analysis of the data was represented by the given Eq. (8):

8 $$\left( {{{\bf{F}}_{\bf{0}}} - {\bf{F}}} \right){\rm{ }} = {\rm{ }}{\bf{5}}.{\bf{938}}{\rm{ }}{\bf{C}}{\rm{ }} + {\rm{ }}{\bf{44}}.{\bf{446}}\;\;\;\;\;\;\;\;\;\;\;\;\;\left( {{\bf{r}}{\rm{ }} = {\rm{ }}{\bf{0}}.{\bf{9990}}} \right)$$

#### Assessment of NIF in human urine

It was not possible to apply the developed method for the determination of NIF in human plasma samples as the method’s sensitivity couldn’t reach its peak plasma concentration (C_max_), therefore, instead, the developed method was successfully applied for its analysis in human urine samples, where about 30–40% of NIF is excreted in the urine unchanged. In patients with normal renal function, average doses of NIF give concentration of about 50.0–200.0 µg/mL (192.0-769.0 µM)^70^. Linear relationship was generated in the urine samples spiked with NIF by plotting its concentrations (µM) vs. the fluorescence quenching (F_0_-F). After data statistical analysis, the mean % recoveries ± SD of the NIF in urine samples were 99.95% ± 1.10 **(**Table 5, **Fig. S4)**. Linear regression analysis of the data was represented by the given Eq. (9):

9 $$\left( {{{\bf{F}}_{\bf{0}}} - {\bf{F}}} \right){\rm{ }} = {\rm{ }}{\bf{5}}.{\bf{1387}}{\rm{ }}{\bf{C}}{\rm{ }} + {\rm{ }}{\bf{336}}.{\bf{23}}\;\;\;\;\;\;\;\;\;\;\;\;\;\left( {{\bf{r}}{\rm{ }} = {\rm{ }}{\bf{0}}.{\bf{9994}}} \right)$$

### Ag-NPs antimicrobial activity

**Fig. 11:**
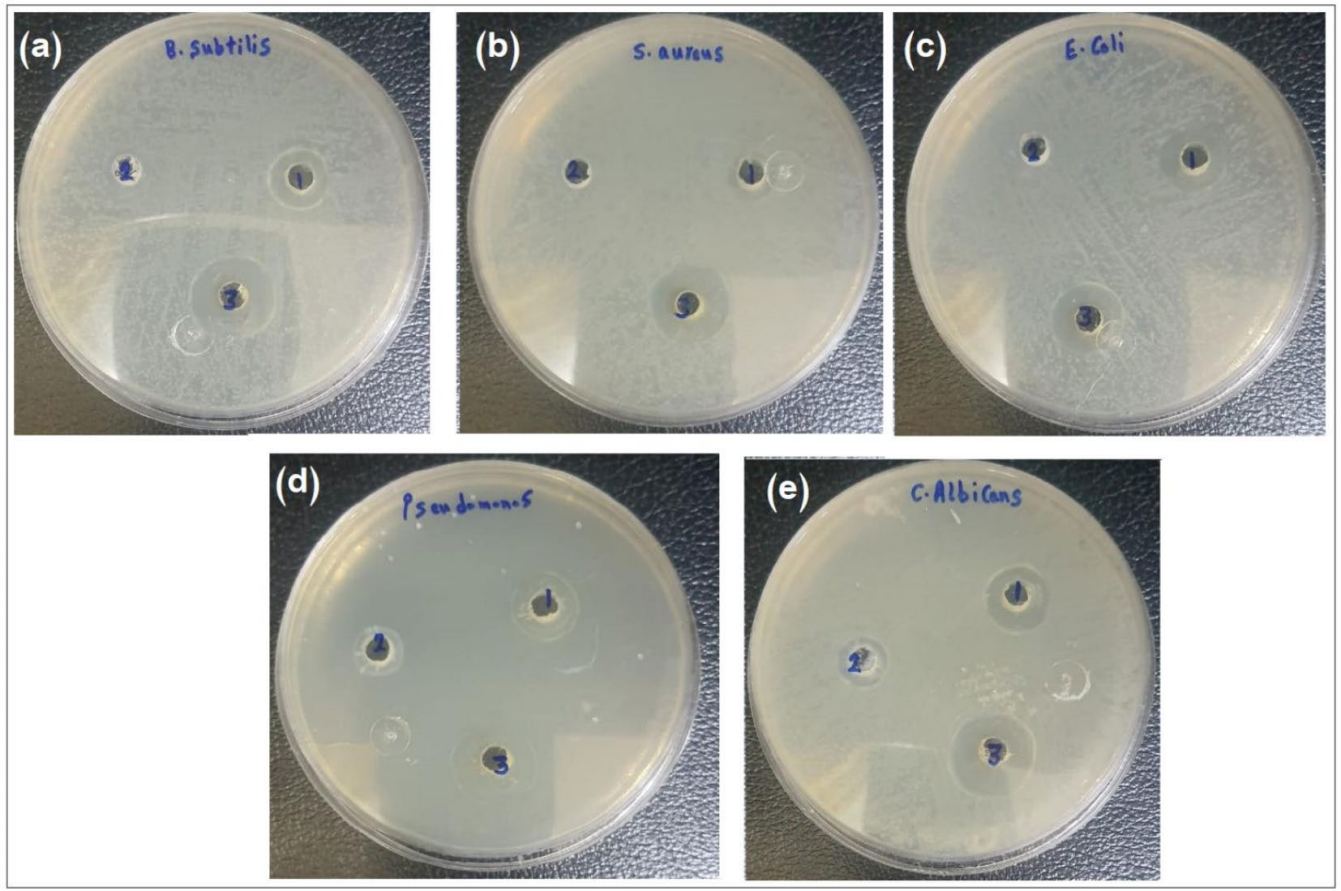
Antimicrobial activity of AgNO_3_ (1), *P. officinalis* extract (2), and Ag-NPs (3), against (**a**) *B. subtilis*, (**b**) *S. aureus*, (**c**) *E. coli*, (**d**) *P. aeruginosa*, and *candida albicans*.

Antimicrobial activity of Ag-NPs was evaluated against various gram-negative and gram-positive bacteria along with *Candida albicans*. Using the agar well diffusion method, inhibition zones were measured revealing that the developed nanoparticles had a noticeable antimicrobial activity over the *P. officinalis* extract and AgNO_3_. Thus, the developed nanoparticles might possess potential applications in antimicrobial therapy (Fig. 11, **Table S3**).

### Greenness assessment

**Fig. 12:**
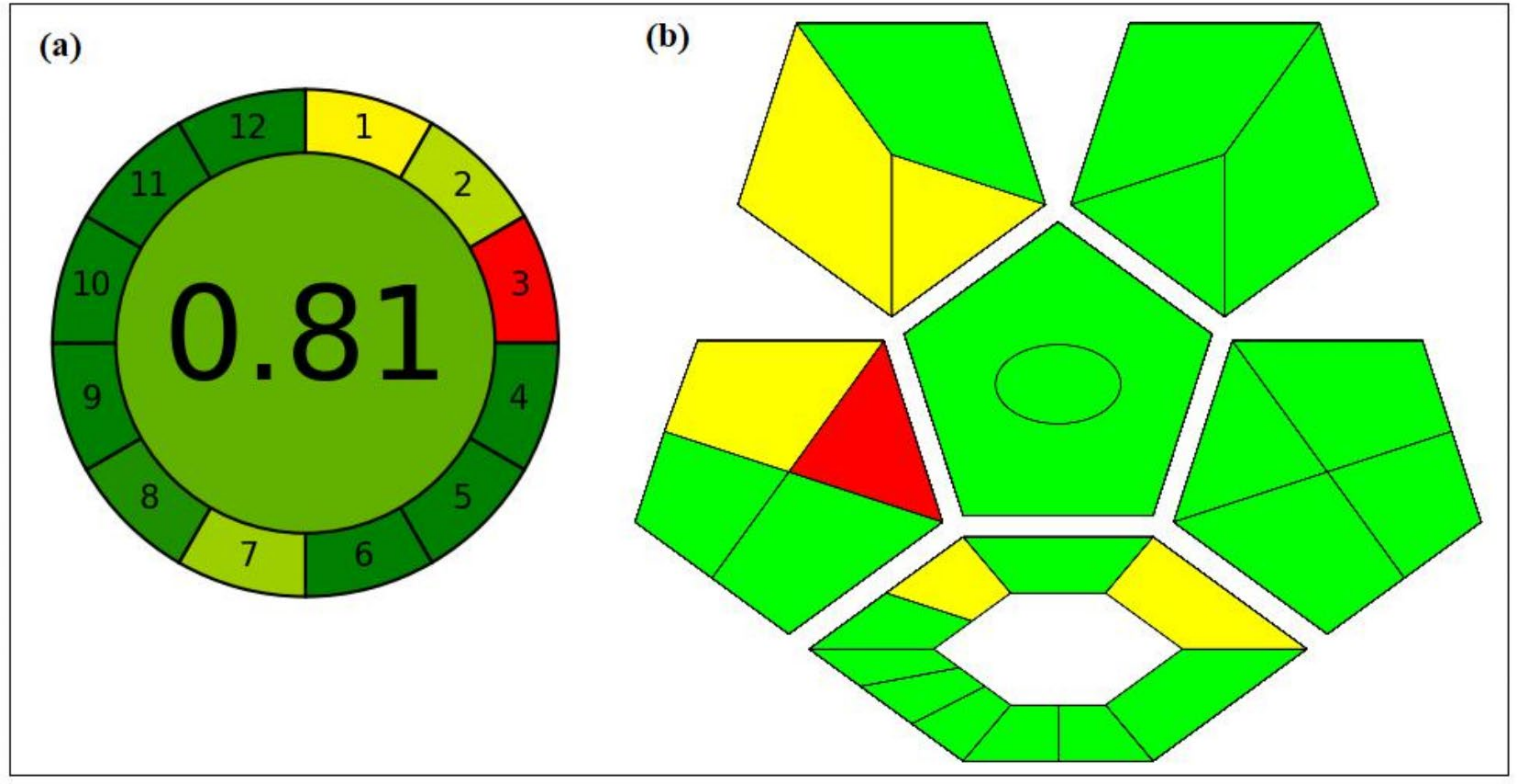
Assessment of the greenness profile of the proposed method by AGREE (**a**) and Complex GAPI (**b**) metrics.

The current method’s greenness was evaluated using the AGREE^72^ and Complex GAPI^73^. AGREE is a metric system utilized for the environmental and occupational threats determination in the developed method. It evaluates 12 critical environmental principles and gives score from 0 to 1 to indicate the greenness of the analytical method. On the other hand, Complex GAPI is a greenness evaluation tool measures the eco-friendly of the developed method by giving colors from red through yellow to green depicting low, medium to highly green method, respectively. It is an advanced version of GAPI that measures the method greenness and the preceding processes, as the synthesis of nanomaterials. In the developed method, fabrication of Ag-NPs was done by the microwave, using a plant extract (*P*. *officinalis*) rather than hazardous chemicals. The developed method used also green reagents and solvents as buffers and distilled water for the estimation of ISN and NIF. Consequently, the method was observed to be excellent green with an AGREE score of 0.81, and green Complex GAPI pictogram, as demonstrated in Fig. 12.

## Conclusion

In this study, a green, straightforward, and quick spectrofluorimetric method was established for the ISN and NIF estimation. The green method utilized the microwave for the preparation of Ag-NPs in only 40 s. The method also used *P. officinalis* root extract for the first time as a reducing agent. Several approaches were utilized for the characterization of the prepared nanoparticles. The proposed method is the first spectrofluorimetric technique that made use of Ag-NPs for the ISN and NIF estimation since the native fluorescence of Ag-NPs was quantitatively quenched following addition of the aforementioned drugs. Different mechanisms of Ag-NPs fluorescence quenching were also investigated, and method validation revealed linearity range of 20.0-100.0 µM and 10.0–60.0 µM for ISN and NIF, respectively. The developed method was accurate, precise, and sensitive. The suggested method was successfully employed for the ISN and NIF estimation in their biological samples and pharmaceutical formulations. The proposed method enhances analytical efficiency while conforming to green chemistry principles, offering an appropriate solution for the analysis of the specified pharmaceuticals. A minor limitation is that the approach may need the optimization of sample preparation procedures to mitigate possible interferences. In the future, the use of advanced sample preparation techniques such as solid-phase extraction and microextraction may substantially improve the sensitivity, efficiency, and application of the established approach. This work connects fluorescence probing with pharmaceutical analysis, providing innovative methods for the quick and precise identification of specified medicines in diverse matrices, addressing the advancing requirements of analytical and bioanalytical chemistry.

## Electronic supplementary material

Below is the link to the electronic supplementary material.

Supplementary Material 1

## Data Availability

The datasets generated and/or analyzed during the current study are available from the corresponding author on reasonable request.
