# Supplementary material for: Ultrafast green synthesis of silver nanoparticles as fluorescent nanosensors for determination of isoniazid and nitrofurantoin in biological fluids and pharmaceuticals
Source: Sci Rep. 2025 May 3;15:15548. doi: 10.1038/s41598-025-98373-6 (PMC12049529; doi:10.1038/s41598-025-98373-6)
Supplement: Supplementary file 1 — Supplementary Material 1 [file 41598_2025_98373_MOESM1_ESM.docx]

**Supplementary Material**

**Ultrafast green synthesis of silver nanoparticles as fluorescent nanosensors for determination of isoniazid and nitrofurantoin in biological fluids and pharmaceuticals**

**Galal Magdy^1,2*^, Eman Aboelkassim^1^, Fathalla Belal^3^**

^1^ Pharmaceutical Analytical Chemistry Department, Faculty of Pharmacy, Kafrelsheikh University, Kafrelsheikh, 33511, Egypt

^2^ Department of Pharmaceutical Analytical Chemistry, Faculty of Pharmacy, Mansoura National University, Gamasa, 7731168, Egypt

^3^ Pharmaceutical Analytical Chemistry Department, Faculty of Pharmacy, Mansoura University, Mansoura, 35516, Egypt

***Corresponding author:** Galal Magdy

**E-mail address:** [galal_magdy@pharm.kfs.edu.eg](mailto:galal_magdy@pharm.kfs.edu.eg)

**Tel.:** +201000137394

**Supplementary Figures captions**

| **Fig. S1** UV-visible absorption spectrum of AgNO_3_ (a) and Ag-NPs (b) |
| --- |
| **Fig. S2** Particle size distribution of Ag-NPs (a) and zeta potential (b) |
| **Fig. S3** Fluorescence emission spectra of the Ag-NPs in spiked human plasma samples upon the addition of different concentrations of ISN (from top to bottom:0.0, 25.0, 35.0, 40.0, 50.0 μM) |
| **Fig. S4** Fluorescence emission spectra of the Ag-NPs in spiked human urine samples upon the addition of different concentrations of NIF (from top to bottom:0.0, 30.0, 40.0, 50.0, 60.0 μM) |

**
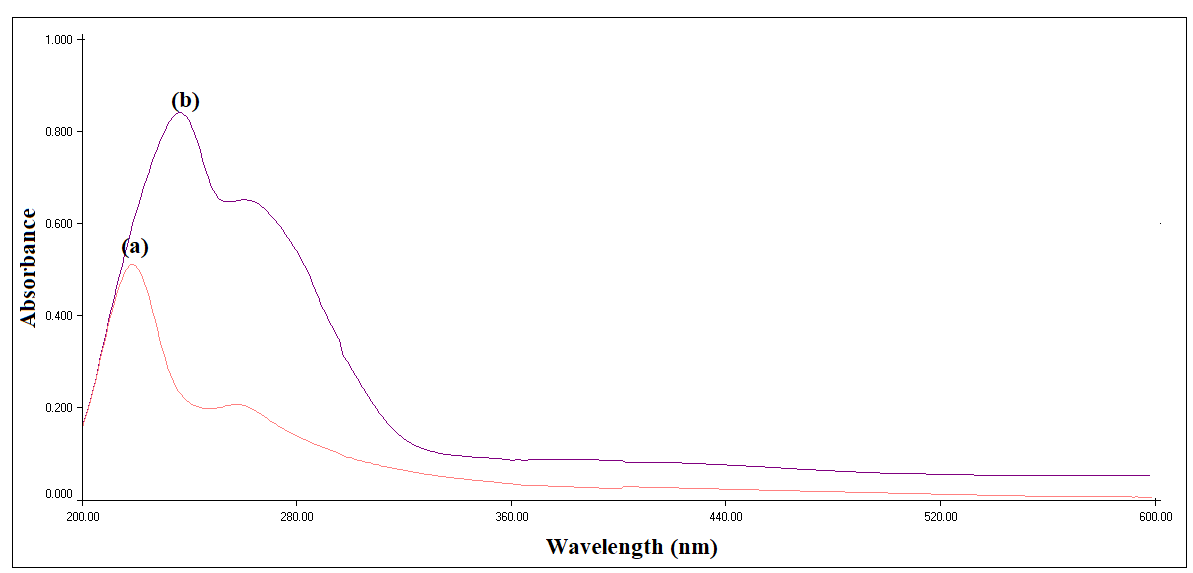
**

**Fig. S1**

**
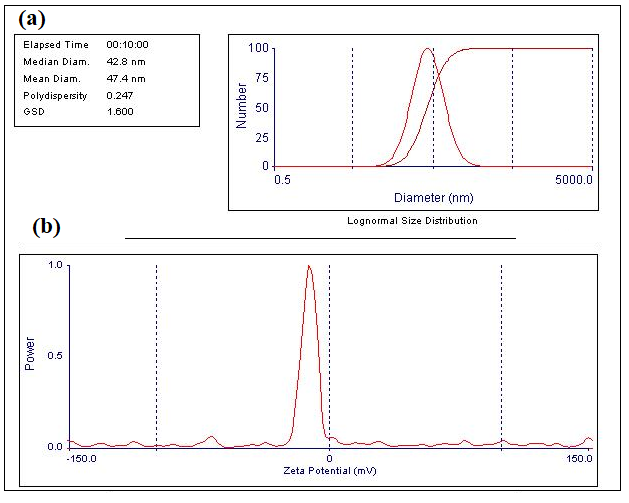
**

**Fig. S2**


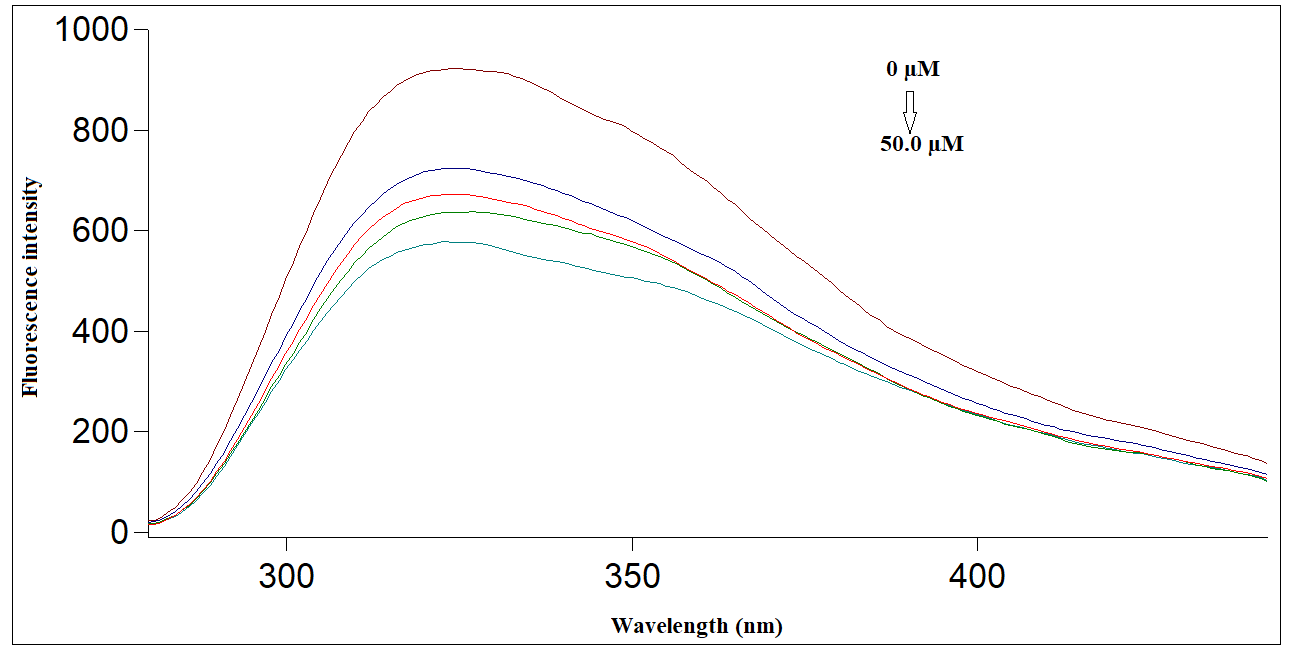


**Fig. S3**

**
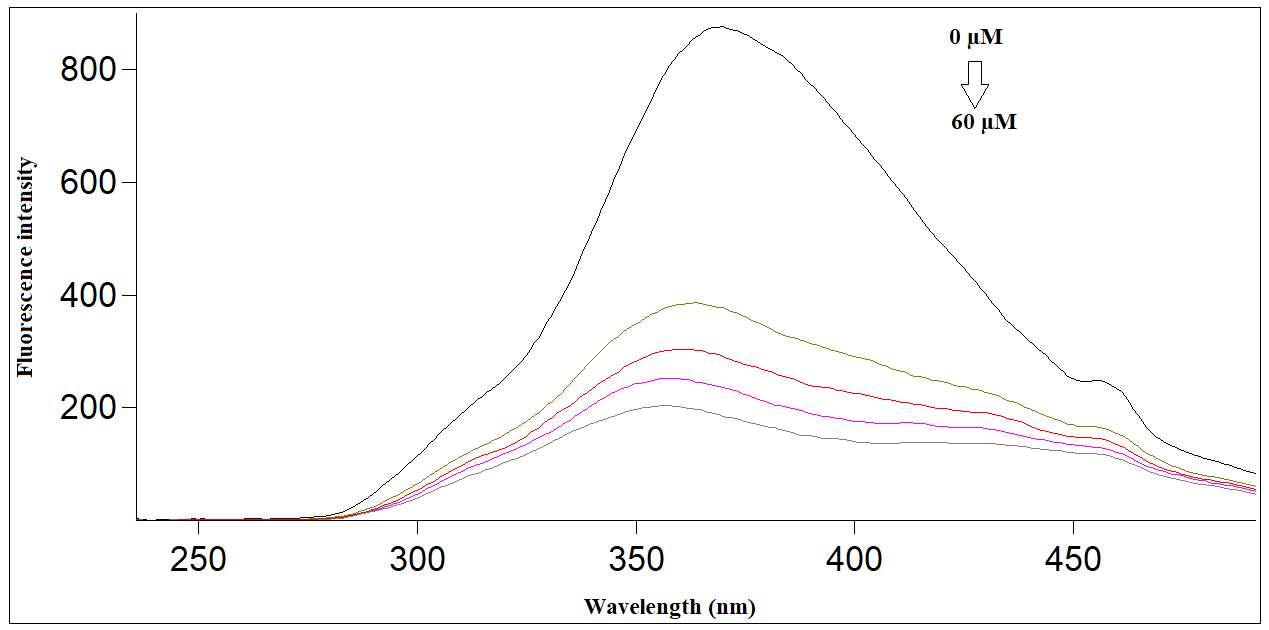
**

**Fig. S4**

**Table S1: Intra-day and inter-day precision data for the determination of the studied drugs by the proposed method**

| **Analyte** | **Conc.**  **taken (µM)** | **Intra-day precision** | | | **Inter-day precision** | | |
| --- | --- | --- | --- | --- | --- | --- | --- |
|  |  | **Conc. found^a^**  **± S.D (μM)** | **% RSD** | **% error** | **Conc. found^a^ ± S.D (μM)** | **% RSD** | **% error** |
| **ISN** | 40.0 | 39.75±0.68 | 0.68 | 0.39 | 39.45±0.52 | 0.53 | 0.31 |
|  | 60.0 | 60.38±0.10 | 0.10 | 0.06 | 60.44±0.37 | 0.38 | 0.22 |
|  | 80.0 | 79.55±0.74 | 0.74 | 0.43 | 79.95±0.32 | 0.32 | 0.18 |
| **NIF** | 30.0 | 30.25±0.24 | 0.24 | 0.14 | 30.24±0.10 | 0.10 | 0.06 |
|  | 40.0 | 40.23±0.37 | 0.36 | 0.21 | 40.33±0.24 | 0.23 | 0.14 |
|  | 50.0 | 50.35±0.31 | 0.31 | 0.18 | 50.36±0.29 | 0.29 | 0.17 |

^a^ Each result is the average of three separate determinations.

**Table S2:** **Robustness evaluation of the proposed method**

| **Factor** | **ISN** | |
| --- | --- | --- |
| 1. **Volume of Ag-NPs (100 μL ± 1)** | **% Recovery** | **%RSD** |
| **95 μL** | 101.03 | 0.50 |
| **100 μL** | 100.55 | 0.22 |
| **105 μL** | 100.75 | 0.25 |
| **Factor** | **NIF** | |
| 1. **Volume of Ag-NPs (100 μL ± 5)** | **% Recovery** | **%RSD** |
| **95 μL** | 100.90 | 0.15 |
| **100 μL** | 100.91 | 0.21 |
| **105 ΜL** | 100.95 | 0.23 |
| 1. **Britton-Robinson buffer pH (10 ± 0.1)** | **% Recovery** | **%RSD** |
| **pH = 9.9** | 101.07 | 0.08 |
| **pH = 10** | 100.91 | 0.21 |
| **pH = 10.1** | 101.10 | 0.1 |
| **3- Volume of Britton-Robinson buffer (1 mL ± 0.1)** | **% Recovery** | **%RSD** |
| **0.9 mL** | 101.12 | 0.08 |
| **1 mL** | 100.91 | 0.21 |
| **1.1 mL** | 101.00 | 0.10 |

**Table S3: Antimicrobial activity of Ag-NPs using agar well diffusion method**

|  | **Inhibition zone diameter (mm)** | | | | |
| --- | --- | --- | --- | --- | --- |
|  | ***S. aureus*** | ***B. subtilis*** | ***P. aeruginosa*** | ***E. coli*** | ***Candida albicans*** |
| **AgNO_3_ (1mM)** | 8 | 12 | 10 | 9 | 13 |
| ***P. officinalis* extract** | 0 | 5 | 4 | 0 | 8 |
| **Ag-NPs** | 14 | 17 | 15 | 12 | 19 |
